# Automated assessment of Knosp grade from pituitary adenoma MRI: Experimental comparison of a rule-based and a machine learning-based approach

**DOI:** 10.1016/j.bas.2026.106117

**Published:** 2026-06-02

**Authors:** Martin Černý, Filip Oplt, Josef Malík, Martin Májovský, Vojtěch Sedlák, Jana Ježková, Miluláš Kosák, Michal Kršek, Václav Hána, Kateřina Sajfrídová, David Netuka, Jan Kybic

**Affiliations:** a Department of Neurosurgery and Neurooncology, Military University Hospital, Prague, Czech Republic; b 1st Faculty of Medicine, Charles University, Prague, Czech Republic; c Department of Cybernetics, Faculty of Electrical Engineering, Czech Technical University, Prague, Czech Republic; d Department of Radiodiagnostics, Military University Hospital, Prague, Czech Republic; e Department of Internal Medicine, Military University Hospital, Prague, Czech Republic; f Third Department of Internal Medicine, First Faculty of Medicine, Charles University and General University Hospital, Prague, Czech Republic

**Keywords:** Pituitary adenoma, Knosp grading- cavernous sinus invasion, Gross total resection, Endocrinological remission, Endoscopic endonasal transsphenoidal surgery

## Abstract

**Background:**

The Knosp grading system is widely used to characterize parasellar extension of pituitary adenomas and to stratify the risk of cavernous sinus (CS) invasion, gross total resection (GTR), and endocrinological remission (ER). However, its assessment relies on expert interpretation of MRI and shows limited inter-rater reliability.

**Objective:**

To develop and compare two automated approaches for Knosp grade assessment from preoperative MRI—one rule-based method emulating the original geometric algorithm and one statistical deep learning–based method—and to evaluate their accuracy and ability to stratify CS invasion, GTR, and ER.

**Methods:**

A geometry-based algorithm was implemented using tumor and internal carotid artery segmentations, generated either manually or automatically. In parallel, a deep learning classifier was trained on 394 annotated MRI scans. Both methods were evaluated on an independent validation cohort of 99 scans. Two additional expert raters independently assigned Knosp grades to assess human inter-rater reliability.

**Results:**

Human raters achieved accuracies of 64.65% (κ = 0.538) and 60.10% (κ = 0.463). The geometry-based method reached 44.95% accuracy (κ = 0.270) with manual segmentations and 35.35% (κ = 0.164) with automatic segmentations, while the deep learning estimator achieved 41.92% (κ = 0.234). Higher Knosp grades assigned by automated methods were significantly associated with increased CS invasion risk and reduced likelihood of GTR (p < 0.05).

**Conclusion:**

Automated approaches can support Knosp grade assessment, but their current accuracy is insufficient for standalone clinical use.

## Highlights

• Automated Knosp grading was evaluated using rule-based and deep learning approaches.
• Rule-based geometric assessment outperformed deep learning in grading accuracy.
• Interrater agreement among human experts remained only moderate.
• Automated Knosp grades stratified risk of cavernous sinus invasion and resection.
• Current automated methods are supportive but not ready for standalone clinical use.

## 1 Introduction

Pituitary adenomas (PAs) are benign tumors located in the sellar region, capable of negatively impacting a patient's quality of life through compression of adjacent structures or dysregulated hormonal secretion (Daly and Beckers, 2020). Transsphenoidal endoscopic surgery is considered the primary treatment for most cases of symptomatic PAs (Molitch, 2017).

Despite the advancements in surgical technique and available equipment, serious postoperative complications can still occur, in particular cerebrospinal fluid leak, diabetes insipidus, meningitis or injury to the internal carotid artery (ICA) or the optic nerve (Chowdhury et al., 2014). Extrasellar invasive growth and cavernous sinus (CS) invasion have been demonstrated to be risk factors for surgical complications and incomplete removal of the tumor (Heffernan et al., 2022).

Several attempts have been made to classify PA growth patterns and their extrasellar extensions. Hardy classification (Hardy and Vezina, 1976), consisting of grades 0-4, introduced a distinction between sellar enlargement (grades 0 - 2) and sellar erosion (grades 3 - 4). A modification of this system was proposed by Wilson et al. (Wilson, 1984), accounting for different types of extrasellar extensions. These classifications have been proposed before the widespread availability of magnetic resonance imaging (MRI).

**Fig. 1:**
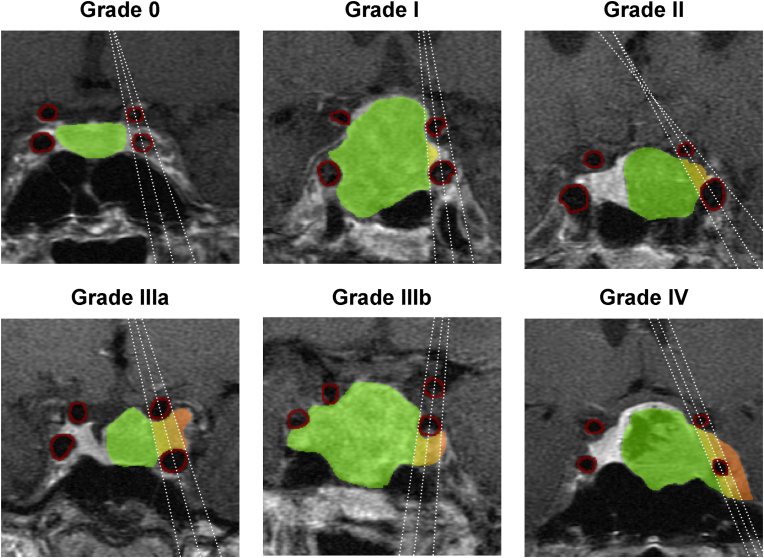
Magnetic Resonance Imaging (MRI) scans demonstrating the Knosp classification of pituitary adenomas, with varying degrees of tumor extension and invasion into the cavernous sinus. The scans are segmented to highlight the tumor in green and the internal carotid artery in red. The progression of grades from 0 to IV reflects the increasing level of tumor invasion, with Grades IIIa and IIIb indicating different patterns of growth in relation to the carotid artery. The medial tangent, connecting line of the centers and the lateral tangent are displayed for the ICA cross-sections on the right side.

In 1993, Knosp et al. devised a classification system aiming to stratify the risk of CS invasion based on preoperative unenhanced and gadolinium-enhanced coronal MRI scans (Knosp et al., 1993). The Knosp score is based on the extent of tumor extension past several defined lines (medial tangent of ICA cross-sections, a line connecting the centers of ICA cross-sections, lateral tangent of ICA cross-sections) and divides PAs into 5 grades (Fig. 1). Micko et al. later subdivided Grade 3 into 3A and 3B, depending on whether the tumor extends above (3A) or below (3B) the intracavernous portion of the ICA, as these two subtypes correlate with significant differences in patient outcomes (Micko et al., 2015).

Recently, the Zurich Pituitary Score was introduced (Serra et al., 2018; The Zurich Pituitary Score predicts). This quantitative score is defined as the ratio of the maximum horizontal tumor diameter to the minimum intercarotid distance at the intracavernous horizontal segment of the ICA. It has shown better interrater agreement than the Knosp score.

**Fig. 2:**
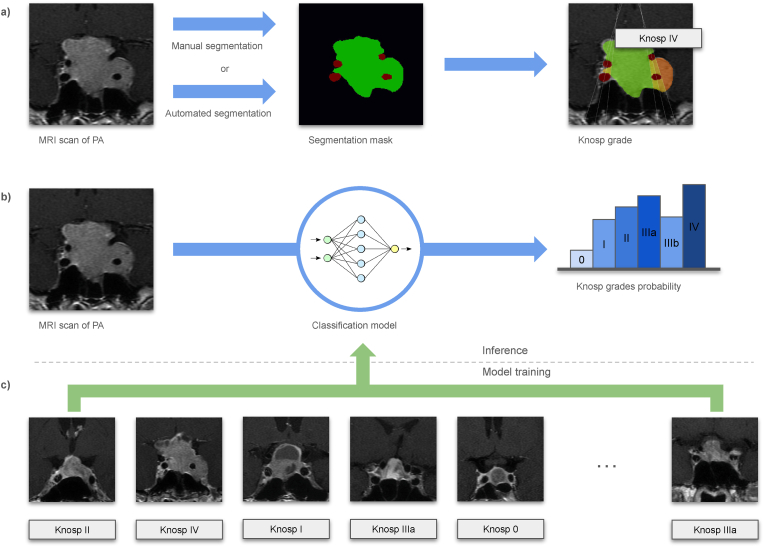
Workflow diagrams illustrating the two automated approaches for assessing the Knosp grade of pituitary adenomas from MRI scans. Panel a) details the geometry-based method utilizing a segmentation mask created through manual or automated processes, followed by algorithmic Knosp grade determination. Panel b) illustrates the machine learning-based method, using an MRI scan as an input to a classification model that computes the probabilities for each Knosp grade. Panel c) showcases a series of annotated MRI scans with manually assigned Knosp grades which are utilized to train the deep learning-based direct estimator (green arrow).

The most widely used classification of PA growth patterns is the Knosp score. The aim of this study is to develop an automated method of Knosp grade assessment from preoperative MRI scans. We demonstrate and experimentally compare two different approaches (Fig. 2).

A geometry-based method follows the definition of lines between the ICA cross-sections determining tumor grade. This approach requires a segmentation of tumor and ICAs, created either manually or automatically (Da Mutten et al., 2024). Encoding *a priori* knowledge of the mechanism of Knosp grade assessment, it represents an algorithmic rule-based method (Haas et al., 2021).

A machine learning-based method utilizes a large number of MRI scans and Knosp grade annotations created by a human rater to train a statistical model (Currie et al., 2019). The model returns probabilities of the tumor belonging to each of six possible classes (Knosp grades). Its inner workings are not easily explainable by intuitive rules (Amann et al., 2020). Since deriving the assessment mechanism *a posteriori* from annotated data, it represents a statistical method (Rajula et al., 2020; Rijcken et al., 2022).

## 2 Methods

### 2.1 Data

Dataset containing preoperative MRI data for 493 patients undergoing primary transsphenoidal (TSF) resection of pituitary adenoma collected for our previous study (Černý et al., 2023) was used. A coronal contrast-enhanced (CE) T1-weighted MRI scan and a segmentation mask classifying each voxel as tumor, ICA, normal gland or background, were available for each patient. The original split into training and validation dataset (394:99) was kept (Table 1).

CS invasion, gross total resection (GTR) were extracted from a prospectively managed database for each patient. Endocrinological (hormonal) remission (ER) was assessed retrospectively from outpatient follow up records and was defined as

A. in Cushing's disease, a nadir serum cortisol level <138 nmol/l within 7 days of surgery, before administration of exogenous glucocorticoids.
B. in acromegaly, normal IGF 1 according to sex and age and random GH < 1 μg/l. Assessment was performed 3 months after surgery.
C. in prolactinoma, normal prolactin level (upper limit of normal prolactin range for the method used: PRL < 29.2 μg/l in fertile women, PRL < 20.3 μg/l in postmenopausal women and PRL < 17.7 μg/l in men).
D. In the case of a pituitary adenoma secreting thyrotropin, normal FT4, FT3 and suppressed or normal TSH within 10 days of surgery.

ER was assessed for functioning adenomas in the validation dataset (n = 40), in three cases the follow-up data was not available.

Knosp grade was assessed by a board-certified neuroradiologist with 15 years of experience for left and right side separately. A board-certified neurosurgeon with 11 years of experience in endoscopic pituitary surgery (rater #2) and a board-certified radiologist with 5 years of experience (rater #3) performed an independent Knosp grade assessment on the validation dataset for inter-rater reliability calculation. Fig. 1 presents a comparison of patient baseline characteristics.

This study was approved by the institutional ethical committee (ref. nr. 108/17-9/2022). Data was anonymized upon patient inclusion and treated according to the ethical standards of the Declaration of Helsinki. The requirement of informed consent was waived by the institutional ethical committees due to the study's large-scale retrospective nature and the absence of potential harm to participants.

### 2.2 Geometry-based model

**Fig. 3:**
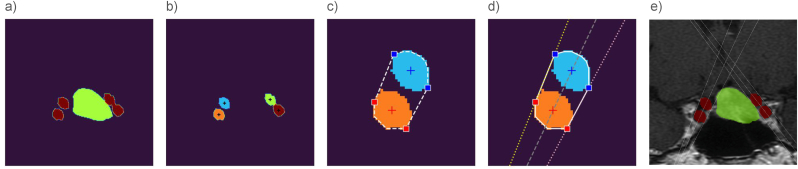
Steps of Knosp grade assessment by the geometry-based method; a) segmentation mask for tumor (green) and ICA (red); b) clustering the segmentation mask for ICA into left and right C3 and C4 segments; c) convex hull of one-sided ICA cross-sections; d) inner and outer tangent and a line connecting the centers of ICA cross-sections; e) classification of individual pixels of the tumor segmentation mask according to their relative position in respect to the drawn lines.

Geometry-based Knosp grade assessment was performed for each slice and side independently (Fig. 3). The maximum value for all slices was considered as the final Knosp grade for the patient. Knosp grades were evaluated independently for manually created segmentations and for segmentations predicted by the model developed in our previous study (Černý et al., 2023).

Pixels representing ICA cross-sections were clustered into four groups using the k-means algorithm (Lloyd, 1982) to find the four cross-sections in each slice. K-means++ initialization with 10 repetitions of the clustering procedure was used to mitigate the risk of encountering a local minimum. The clusters were then assigned to the left and right cavernous (C3) and supraclinoid (C4) segments of the ICA based on the relative positions of the centers of mass (COM) of the cross-sections.

Lines connecting the COMs of cross-sections on one side were drawn. A convex hull of both cross-sections of the ICA on one side was determined and expressed as a polygon. Pairs of subsequent vertices of the convex hull belonging to different segments of the ICA determined the tangents of both ICA segments.

The position of pixels of the tumor segmentation mask with respect to the drawn lines was then determined. A tolerance of a half pixel is imposed so that points lying up to 0.5 pixels far from the line were considered to lay directly on the line and not to increase the grade. For pixels laying past the lateral tangent, their position relative to the horizontal line through the C3 cross-section COM was examined to classify them as either grade 3a or 3b. Tumors that completely surround the cross-section were considered grade 4.

The code is available from the authors' Github repository (Černý and Oplt, 2024).

### 2.3 Machine learning model

To directly estimate the Knosp score from the images, a custom modification of the ResNet18 model was employed (He et al., 2016). The original convolutional blocks from ResNet18 were retained, while the classification head was replaced with a custom architecture consisting of a global average pooling layer to reduce the spatial dimensions of the convolutional output and of two dense layers, the first with 1000 units and ReLU activation function and the later with 6 units corresponding to the number of classes (Knosp grades) in the dataset and a softmax activation function (Yosinski et al., 2014). The output layer provided predicted probabilities for each class, with the class corresponding to the highest probability selected as the model's final prediction.

The model was initialized with pre-trained ResNet18 wt, and all layers were set as trainable. It was compiled using the Adam optimizer with an initial learning rate of 0.001 and categorical cross-entropy loss (Kingma and Ba, 2015). The model was trained on a GPU computation node with NVIDIA Tesla V100 GPU for a maximum of 250 epochs, with early stopping applied if no improvement was observed after 20 consecutive epochs (Prechelt et al., 1998).

Data augmentation consisted of random rotations between −15 and + 15°, random horizontal and vertical shifts of up to 10% of the image dimensions, and random zooming in and out by up to 15% (Garcea et al., 2023). Missing regions resulting from these transformations were filled using the nearest pixel values from the image edge. Subsequently, sample normalization was applied to ensure a mean of 0 and a standard deviation of 1 for each sample (Reinhold et al., 2019).

The model predicted Knosp score for the left side only. To obtain predictions for the right side, a flipped image was fed to the model. This way, we avoided training two classifiers with an identical task for both left and right side and achieved further data augmentation by using each side as an independent sample.

### 2.4 Statistical evaluation

The per-slice predictions from the evaluated algorithms were converted to per-patient per-slice Knosp scores as the highest grade among all slices for respective sides. Accuracy and Cohen's Kappa coefficient were computed to assess the agreement between the automated methods and the ground truth and raters #2 and #3. Confusion matrices were generated for a detailed comparison of the performance of each method. The Spearman's rank correlation coefficient (ρ) was calculated to test for a correlation between the Knosp grade and the likelihoods of CS invasion, GTR and ER. For the purpose of the risk stratification analysis, grades IIIa and IIIb were merged together due to the low prevalence of grade IIIb tumors. Source data for the statistical evaluation are available as Supplementary 1.

## 3 Results

### 3.1 Deep learning-based direct estimator training

The model achieved highest prediction accuracy after 52 epochs after which there was no further improvement for another 20 epochs and early stopping was triggered. The training took 6 min 13 and seconds. The trained model is available from the authors' Github repository (Černý and Oplt, 2024).

### 3.2 Performance of individual methods

**Fig. 4:**
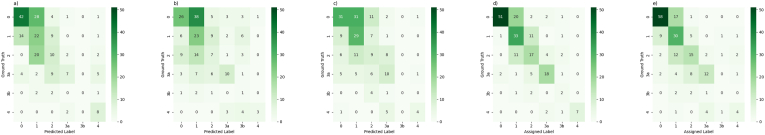
Confusion matrices for a) geometry-based method on manually created segmentations, b) geometry-based method on automatically predicted segmentations, c) machine learning-based method, d) human rater #2 and e) human rater #3.

The human raters achieved accuracies of 64.65% (Cohen's kappa = 0.538, one-grade tolerance accuracy (1gta) of 91.92%) for rater #2 and 60.10% (κ = 0.463, 1gta = 87.88%) for rater #3. The geometry-based method on manual segmentations achieved 44.95% (κ = 0.270, 1gta = 86.87%), while on predicted segmentations it reached 35.35% (κ = 0.164, 1gta = 75.25%). The deep learning-based direct estimator showed accuracy of 41.92% (κ = 0.234, 1gta = 78.79%). Fig. 4 presents confusion matrices for individual methods.

### 3.3 Risk stratification

**Fig. 5:**
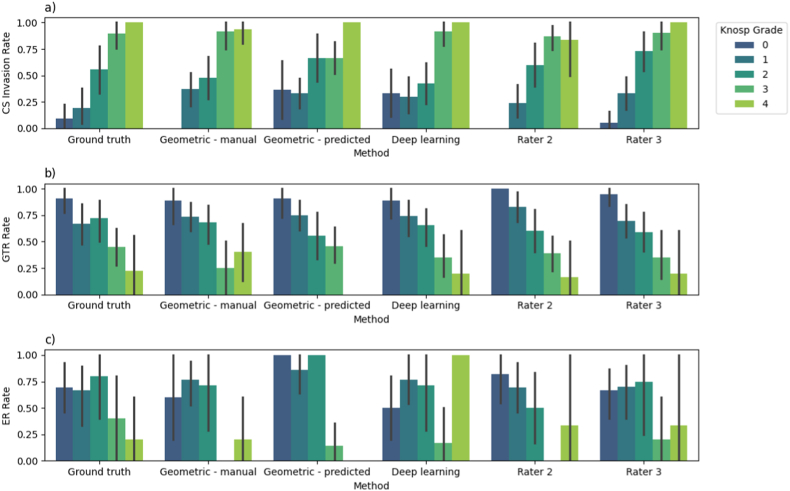
Comparative analysis of Knosp grade assessment methods across clinical outcomes, represented in bar graphs. Panel a) displays the CS invasion rate, panel b) the GTR rate, and panel c) the ER rate, across varying Knosp grades (0, 1, 2, 3, and 4). Each method—ground truth, geometric with manual segmentation, geometric with automatic segmentation, deep learning-based direct estimator, and a human rater #2 and #3—is evaluated for its predictive accuracy against these clinical metrics. Error bars indicate variability or confidence intervals within each assessment category.

A higher Knosp grade was associated with an increased risk of CS invasion across all assessment methods, with a statistically significant correlation observed for the ground truth, geometric assessment from manual segmentations, the deep learning-based direct estimator, and raters #2 and #3 (p < 0.05). Similarly, higher Knosp grades corresponded to a decreased likelihood of GTR for the ground truth and all assessment methods (p < 0.05). For ER, only the Knosp grades assigned by rater #2 demonstrated a significant negative correlation with ER likelihood (p = 0.037). Table 2 presents detailed likelihoods of CS invasion, GTR, and ER stratified by Knosp grade for each assessment method. Fig. 5 offers a graphical comparison of the likelihoods for CS invasion, GTR, and ER across Knosp grades assigned by each method.

**Table 1:** Baseline characteristics of the patient dataset. Significance of the difference between the training and validation dataset is calculated using the unpaired *t*-test for age and the chi-square test for categorical variables. ∗Data on endocrinological remission was collected retrospectively for functioning adenomas in the validation dataset (n = 40).

	Total	Train	Validation	Difference, *(p-value)*
*N*	493	394	99	
Age, *years ± SD*	53.4 ± 15.5	53.2 ± 15.6	54.2 ± 14.8	0.556
Male sex, *n (%)*	236 (47.9)	179 (45.4)	57 (57.6)	0.040
Tumor type, *n (%)*				
Non-functioning	301 (61.1)	242 (61.4)	59 (60)	0.828
GH-secreting	120 (24.3)	99 (25.1)	21 (21)	0.496
Prolactin-secreting	17 (3.4)	12 (3)	5 (5)	0.503
ACTH-secreting	53 (10.8)	39 (9.9)	14 (14)	0.300
Plurihormonal	3 (0.6)	3 (0.8)	0 (0)	0.882
Knosp grade, *n (%)*				
Grade 0	73 (14.8)	55 (14.0)	18 (18.2)	0.369
Grade I	146 (29.6)	118 (29.9)	28 (28.3)	0.840
Grade II	108 (21.9)	87 (22.0)	21 (21.2)	0.959
Grade IIIa	97 (19.7)	81 (20.6)	16 (16.2)	0.400
Grade IIIb	15 (3.0)	12 (3.0)	3 (3.0)	0.749
Grade IV	54 (11.0)	41 (10.4)	13 (13.1)	0.551
CS invasion, *n (%)*	266 (54.0)	215 (54.6)	51 (51.6)	0.666
GTR, *n (%)*	302 (61.3)	240 (60.9)	62 (62.6)	0.844
ER *n (%)*			22/37∗ (59.5)

**Table 2:** Rates of CS (parasellar) invasion, gross total resection, and endocrinological remission (%) stratified by Knosp grade for each assessment method, correlation between Knosp grade and likelihood of CS invasion is expressed using the Spearman's rank correlation coefficient (ρ) and p-value. Rates reported in the original studies [7,8] are shown for comparison (these were derived from separate patient cohorts with possibly varying distributions). ∗ - unclear because of scars from a previous surgery, could possibly be 12.5% (1 out of 8 cases).

	Ground truth	Geometry - manual segmentation	Geometry - automatic segmentation	Deep learning-based estimator	Rater #2	Rater #3	Knosp [7]	Micko [8]
Cavernous sinus (CS) invasion rates								
**Grade 0**	9.1	0	36.4	33.3	0	5.3	0∗	
**Grade I**	19	36.8	33.3	29.6	24.1	33.3	12.5	1.5
**Grade II**	55.6	48	66.7	42.3	60	72.7	87.5	9.9
**Grade III**	89.7	91.7	66.7	91.3	87.1	90	85.7	IIIa - 26.5 IIIb - 70.6
**Grade IV**	100	93.3	100	100	83.3	100	100	100
**CS invasion likelihood correlation**	ρ = 1.0 p < 0.001	ρ = 1.0 p < 0.001	ρ = 0.872 p = 0.054	ρ = 0.9 p = 0.037	ρ = 0.9 p = 0.037	ρ = 1.0 p < 0.001		
**Gross total resection (GTR) rates**								
**Grade 0**	90.9	88.8	90.9	88.9	100	94.7		
**Grade I**	66.7	73.7	75	74	82.8	69.7		83
**Grade II**	72.2	68	55.6	65.4	60	59.1		71
**Grade III**	44.8	25	45.5	34.8	38.7	35		IIIa - 85 IIIb - 64
**Grade IV**	22.2	40	0	20	16.7	20		0
**GTR likelihood correlation**	ρ = −0.9 p = 0.037	ρ = −0.9 p = 0.037	ρ = −1.0 p < 0.001	ρ = −1.0 p < 0.001	ρ = −1.0 p < 0.001	ρ = −1.0 p < 0.001		
**Endocrinological remission (ER) rates**								
**Grade 0**	69.2	60	100	50	81.2	66.7		
**Grade I**	66.7	76.5	85.7	76.9	69.2	70		88
**Grade II**	80	71.4	100	71.4	50	75		60
**Grade III**	40	0	14.3	16.7	0	20		IIIa - 67 IIIb - 0
**Grade IV**	20	20	0	100	33.3	33.3		0
**ER likelihood correlation**	ρ = −0.7 p = 0.188	ρ = −0.6 p = 0.285	ρ = −0.821 p = 0.089	ρ = 0.3 p = 0.624	ρ = −0.9 p = 0.037	ρ = −0.5 p = 0.391

## 4 Discussion

The study compared two automated methods for Knosp grade assessment in terms of their accuracy and reliability. Our results demonstrate the potential of automated tools to reduce interobserver variability and improve consistency in the evaluation of pituitary adenomas. However, the accuracy achieved is still insufficient for clinical use.

One of the primary advantages of both methods is their ability to provide consistent and objective Knosp grade assessments. Automated systems eliminate the subjective interpretation inherent in manual grading, thereby standardizing the evaluation process. This feature is particularly important given the variability in human assessments, as previously criticized in literature (Mooney et al., 2017). The data presented in this study confirmed the poor interrater reliability even among experienced human raters (accuracy of 64.65% and 60.10%, Cohen's kappa of 0.538 and 0.463).

In the future, similar systems could be integrated into healthcare information infrastructures to automate the analysis of patient data (Botwe et al., 2021). These systems could offer significant time savings by performing tasks that would otherwise require expert input. In busy clinical settings, the ability to quickly generate reports from preoperative MRI scans could allow neurosurgeons to focus on higher-level decision-making and treatment planning (Iqbal et al., 2022; Noh et al., 2023). Similar systems could also guide other specialists treating pituitary adenoma patients, such as endocrinologists, who may not be accustomed to evaluating imaging data. This would enable more informed discussions with patients about surgical risks and expected outcomes, further improving the precision and personalization of complex multidisciplinary care for pituitary patients (Grayson et al., 2021).

However, it is important to emphasize the need for careful review and interpretation of imaging data, particularly given the complex anatomical details they visualize. While the Knosp score is a valuable tool for assessing CS invasion and related surgical risks, it is, like any model, a simplification of reality. The question of CS invasion should not be reduced to assigning a grade on a fixed scale; neurosurgeons must consider multiple factors, including the size and consistency of the tumor, its relationship to critical neurovascular structures such as ICA and cranial nerves, the degree of lateral extension into the CS and suprasellar extension, the involvement of the sellar floor, and pre-existing conditions that may influence surgical risk, such as prior surgeries, hormonal activity of the adenoma, and overall patient health (Zada et al., 2011). These elements provide a more comprehensive picture of the risks and help guide the surgeon in formulating a tailored surgical approach and counseling the patient.

The geometry-based method, which replicates the original assessment algorithm, demonstrated higher accuracy and risk stratification ability compared to the machine learning approach. These results suggest that encoding expert knowledge into a deterministic algorithm remains a robust solution, especially when high-quality segmentation masks are available. This finding contrasts with recent trends in artificial intelligence, where statistical models often outperform rule-based methods (Halevy et al., 2009), and most breakthrough advances are linked to the development of new machine learning architectures and larger models (Brown et al., 2020; Kaplan et al.).

We believe this discrepancy could be due to the limited size of our dataset. Much of the recent progress in AI has been driven by the accumulation of large training datasets (Sun et al., 2017). This remains a significant challenge in clinical settings, where contributing factors include the costly labor of highly trained medical professionals required for data annotation, the from an ML perspective relatively low number of patients even in larger centers, and restrictions on data sharing due to its sensitive nature and legal requirements (Ahmed et al., 2023; Youssef et al., 2023). With larger, more diverse training datasets and further refinement of the model architecture, this approach may surpass rule-based systems. Furthermore, machine learning offers the advantage of continuous improvement with more data, whereas the geometry-based method is constrained by its reliance on predefined rules (Kaplan et al.; Sun et al., 2017).

While the Knosp grading system is still widely used, other approaches, such as the Zurich Pituitary Score (ZPS), have been proposed to address some of its limitations (Serra et al., 2018). The ZPS, a more quantitative method, has shown better interrater agreement in previous studies (The Zurich Pituitary Score predicts). However, the simplicity and clinical familiarity of the Knosp grading system make it a practical choice for many neurosurgeons. Another possible direction for future work is extending the geometry-based method into a 3D model, which could account for more complex tumor morphologies and variations in anatomical structures. This could further improve the precision of Knosp grade assessments, especially in borderline cases that challenge traditional 2D evaluations.

It is important to note that Knosp grading, particularly at lower grades (0–II), does not always correspond to histologically confirmed cavernous sinus invasion. The original classification was devised based on preoperative MRI appearance, and subsequent surgical series have shown that radiological encasement of the ICA does not reliably predict intraoperative findings for lower-grade tumors (Knosp et al., 1993; Micko et al., 2015). In the study by Micko et al., endoscopic intraoperative verification revealed that cavernous sinus invasion rates for Knosp grades I and II were only 1.5% and 9.9%, respectively, confirming that lower Knosp grades frequently overestimate the degree of true CS invasion (Micko et al., 2015). This discrepancy between radiological grade and actual invasion may partly explain the lower accuracy observed in our automated methods for lower-grade tumors, as the ground truth itself reflects MRI appearance rather than confirmed histological invasion. This also underscores a fundamental limitation of any MRI-based grading approach and represents a meaningful challenge for automated systems attempting to replicate human rater performance on this task.

While advancements in automated methods, including rule-based algorithms and machine learning models, hold great promise for enhancing the precision and efficiency of pituitary adenoma assessment, much work remains before these tools can outperform traditional evaluation methods. These technologies offer the potential to improve consistency and streamline clinical workflows, but they should be viewed as complementary aids rather than substitutes for expert judgment (Sezgin, 2023). The complexity of anatomical structures and variability in individual cases highlight the crucial role of human oversight—neurosurgeons and radiologists must critically assess automated results within the context of broader clinical knowledge and experience. Ultimately, the integration of these technologies into clinical practice will depend on achieving a balance between the power of automation and the irreplaceable insights of skilled human professionals (Botwe et al., 2021).

## 5 Limitations

All MRI scans and associated clinical data were obtained from a single institution, which may limit the generalizability of the findings to other centers with different imaging protocols or patient demographics.

Ground truth Knosp grades were assigned by a single human rater.

Accuracy of the rule-based method is influenced by the quality of manually created segmentations, potentially biasing the assessment of the performance of the method itself.

Dataset size and distribution may influence the accuracy of the deep learning-based direct estimator, potentially biasing the assessment of the performance of the method itself.

The study used a modified ResNet18 model, which may not capture the full potential of more complex, state-of-the-art architectures, or architectures that will be developed in the future.

Endocrinological remission data was collected retrospectively, which may introduce bias and affect the accuracy of remission assessments.

## 6 Conclusions

Our study demonstrates the potential of automated methods to support Knosp grade assessment for pituitary adenomas, highlighting the consistent and objective performance of a rule-based geometry method over a machine learning-based approach. While the geometry-based model outperformed the deep learning-based direct estimator in accuracy and reliability, neither method achieved the precision required for independent clinical use. Future advancements with larger datasets and optimized model architectures may enhance the capabilities of machine learning, potentially allowing for a hybrid approach that leverages the strengths of both methods. For clinical integration, these tools should be viewed as complementary aids that augment, rather than replace, expert evaluation in complex neurosurgical planning.

## Ethical standards

This study was approved by the institutional ethical committee of Military University Hospital (ref. nr. 108/17-9/2022). Data were anonymized at the time of patient inclusion and treated according to the ethical standards of the Declaration of Helsinki. The requirement of informed consent was waived by the institutional ethical committee because of the retrospective nature of the study and no potential harm to the study participants.

## Declaration of generative AI and AI-assisted technologies in the manuscript preparation process

No AI tools were used in the preparation of this manuscript

## Declaration of competing interest

The authors declare the following financial interests/personal relationships which may be considered as potential competing interests: David Netuka reports financial support was provided by Ministry of Health of the Czech Republic. Martin Cerny reports financial support was provided by Ministry of Health of the Czech Republic. If there are other authors, they declare that they have no known competing financial interests or personal relationships that could have appeared to influence the work reported in this paper.
